# 

**Published:** 1821-03

**Authors:** 


THE LONDON
### Medical and Physical Journal.

3 OF VOL. XLV.] MARCH, 1821. [NO. 265.

### NOTICE.

*Another of the series of* PRŎEMIA *to the several volumes of this Journal, (which commenced with that to the forty-third volume,) comprising a History of the Progress of Medicine and its auxiliary Sciences for the half-year immediately previous to the period of their production, respectively, was published on the last day of January. One of the especial intentions of those* Prŏemia, *is to present a comprehensive view of the state and progress of Medicine throughout Europe generally, and in the United States of America; an object that cannot be effected in the regular monthly Numbers of the Journal, because of the small extent of space which can be there appropriated to this purpose.*

### MONTHLY CATALOGUE OF MEDICAL BOOKS.

A Practical Treatise on Hydrocephalus Acutus, or inflammatory water in the head; by Leopold Anthony Gölis, physician and director of the institution for the sick children of the poor at Vienna, translated from the German by Robert Good, M.D.

A Conspectus of the Pharmacopœias of the London, Edinburgh, and Dublin College of Physicians, being a practical Compendium of Materia Medica and Pharmacy, by Anthony Todd Thompson, F.L.S. Third edition.

A Dissertation on the Treatment of Morbid Local affections of Nerves, to which the Jacksonian prize was adjudged by the Royal College of Surgeons, by Joseph Swan, Member of the Royal College of Surgeons. In 1 vol. 8vo. with three copper-plate engravings. Price 10s. 6d. boards.

A Dissertatio on Infanticide, in its Relations to Physiology and Jurisprudence. By William Hutchinson, M.D. Member of the Society of the College of Physicians of Paris; Fellow of the Linnæan Society; Member of the Medical and Chirurgical Society of London; and one of the Physicians to the Royal Metropolitan Infirmary for Sick Children. Second Edition.

Practical Observations on the Colchicum Autumnale, as a general Remedy of great Power in Disorders which are connected with increased Action of the Heart and Arteries; and therefore as a Substitute for Bleeding in the Treatment of Inflammatory Diseases, both acute and chronic. By Charles Haden. 8vo. 4s.

